# Current patterns of care in low‐risk thyroid cancer—A national cross‐sectional survey of Australian thyroid clinicians

**DOI:** 10.1002/edm2.398

**Published:** 2023-02-03

**Authors:** Winy Widjaja, Christopher W. Rowe, Christopher Oldmeadow, Daron Cope, Elizabeth A. Fradgley, Christine Paul, Christine J. O'Neill

**Affiliations:** ^1^ Surgical Services, John Hunter Hospital Newcastle New South Wales Australia; ^2^ Department of Endocrinology John Hunter Hospital Newcastle New South Wales Australia; ^3^ University of Newcastle Newcastle New South Wales Australia; ^4^ Hunter Medical Research Institute Newcastle New South Wales Australia

**Keywords:** thyroid cancer, health‐related quality of life, thyroidectomy, cancer survivorship, fear of cancer recurrence, multi‐disciplinary team

## Abstract

**Introduction:**

De‐escalated treatment of hemithyroidectomy without radioactive iodine (RAI) is now accepted for patients with low‐risk, well‐differentiated thyroid cancer (WDTC). The benefit of long‐term follow‐up care remains controversial. This study aims to describe parameters associated with less than total thyroidectomy, and discharge from specialist follow‐up in patients with low‐risk WDTC in Australia.

**Methods:**

An online survey was distributed to Australian members of Endocrine Society of Australia, Australian and New Zealand Endocrine Surgeons, and Australian Society of Otolaryngology, Head and Neck Surgery. Clinicians completed a survey of management and follow‐up care preferences for four clinical vignettes (all low‐risk WDTC).

**Results:**

119 clinicians (48% endocrinologists, 55% male) answered at least one question. The majority (59%) of respondents recommended less than total thyroidectomy and omission of RAI in patients with WDTC <2 cm. Most (62%) would discharge a patient with micropapillary thyroid cancer within 1 year following total thyroidectomy. In contrast, for WDTC 1–4 cm, >90% of clinicians would continue specialist follow‐up for at least 5 years. The majority of clinicians felt that patients experienced disproportionate fear of recurrence and were reassured by follow‐up. After multivariable analysis, clinicians who participated in multidisciplinary teams (MDTs) were more likely to choose de‐escalated care for both initial treatment (*p* = .005) and follow‐up care (>5 years, *p* = .05).

**Conclusion:**

Clinician attitudes captured by this survey reflect recent changes in guidelines towards hemithyroidectomy for low‐risk WDTC, particularly amongst MDT attendees. There is a need to further examine the impact of de‐escalated care on fear of recurrence and quality of life in thyroid cancer survivors.

## Graphical abstract

This survey of Australian thyroid clinicians, demonstrates that de‐escalation of surgical care (less than total thyroidectomy) of patients with low‐risk thyroid cancer has become common since the 2015 American Thyroid Association Guidelines were published. Clinicians feel that patients fear of cancer recurrence is disproportionately excessive compared to the true risk; but that they are reassured by clinician follow‐up.

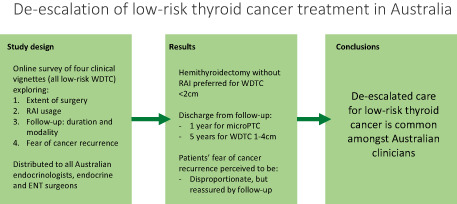

## 1 INTRODUCTION

Well‐differentiated thyroid cancer (WDTC) accounts for more than 95% of thyroid cancer diagnoses. Generally, it carries an excellent prognosis with a 10‐year survival of >97%.^1^, ^2^ This is partly due to early detection and effective surgical and medical treatment, but it is also due to the over‐diagnosis of indolent cancers.^3^ Despite a good prognosis, it has been recently shown that health‐related quality of life (HRQoL) detriments are common in thyroid cancer survivors.^4^, ^5^ The 2015 American Thyroid Association (ATA) management guidelines advocated for a paradigm shift towards the de‐escalation and individualisation of management of low‐risk WDTC.^6^ There is a need for contemporary data regarding the uptake of these guidelines, particularly outside of the United States.

The appropriate length and frequency of specialist follow‐up for WDTC remain uncertain, and few guidelines exist.^7^ This may result in significant heterogeneity in practice, possibly with detriment to both patients and health services. For example, for those with low‐risk disease and excellent response to treatment, longer term follow‐up can be associated with false positive ultrasound results and is unlikely to be cost effective or an appropriate use of healthcare resources.^8^, ^9^ It is also unclear what the impact of de‐escalation of initial care might have on follow‐up practices. Finally, recent evidence has highlighted high rates of anxiety, depression and fatigue in low‐risk thyroid cancer patients, driven in part by high rates of fear of cancer recurrence (FCR).^10^, ^11^ The impact of longer‐term follow‐up on patient HRQoL and FCR is unknown.^12^

The aim of this study was to explore specialists' preferences for uptake of de‐escalation and follow‐up in patients with low‐risk WDTC, with particular reference to the group of patients identified as suitable for treatment with less than total thyroidectomy by the 2015 ATA thyroid cancer guidelines.^6^ We aimed to identify important factors that impact treatment decisions in low‐risk thyroid cancer. These data can facilitate the development of standardised guidelines, highlight potential implementation challenges to de‐escalated care and inform future research into HRQoL in thyroid cancer.

## 2 METHODS

### 2.1 Survey development

This study was prospectively approved by the Hunter New England Human Research Ethics Committee (2020ETH01892) and the University of Newcastle Human Research Ethics Committee (H‐2021‐0058). A 50‐item study‐specific survey was developed by a team of endocrine surgeons; ear, nose and throat (ENT) surgeons, endocrinologists and behavioural scientists (Appendix 1). Survey elements included clinician demographic characteristics (age, gender, geography, medical speciality, years in practice, annual thyroid cancer caseload and MDT attendance). Structured responses to four clinical vignettes (Table 1) investigated initial treatment and follow‐up preferences for patients with very low and low‐risk WDTC according to ATA guidelines.^6^ The scenarios were presented to survey participants in order of increasing risk and aimed to explore clinician's preferences for surgical intervention, long‐term specialist follow‐up, neck ultrasound and time to discharge back to primary care according to patient and tumour characteristics (gender, age, tumour size, lymph node involvement, vascular invasion, BRAF V600E status and extent of surgery). The survey also asked clinicians about their perception of patients' FCR and whether follow‐up care might exacerbate or ameliorate this. The survey was piloted by a group of ten clinicians from different geographical locations across Australia.

**TABLE 1:** Clinical vignettes description.

Scenario 1	A 45‐year‐old female patient has an incidental papillary thyroid carcinoma in the pathology specimen after a total thyroidectomy for a multinodular goitre, the tumour measures 4 mm and there are no other concerning features.
Scenario 2	A 40‐year‐old female patient presents with an incidentally discovered papillary thyroid carcinoma measuring 15 mm. The tumour is completely intra‐thyroidal, the contralateral lobe is normal on ultrasound and there are no suspicious lymph nodes. Pre‐operative TSH is <2 mIU/L.
Scenario 3	A 65‐year‐old female patient has a solitary 2 cm left thyroid nodule with Bethesda IV cytology. The contralateral lobe is normal, and there are no suspicious lymph nodes either clinically or on ultrasound. She undergoes a hemithyroidectomy. The pathology report reveals an 18 mm minimally invasive follicular thyroid cancer with capsular but not vascular invasion.
Scenario 4	A 48‐year‐old male patient presents with a solitary right thyroid nodule. It measures 35 mm and has suspicious sonographic features. Pre‐operative cytology is Bethesda VI, suggestive of papillary thyroid cancer. There are no suspicious cervical lymph nodes either clinically or on ultrasound. The contralateral lobe is normal.

### 2.2 Study population and administration

The cross‐sectional online survey was administered on the REDcap® platform from October 2020 to January 2021.^13^ The survey was sent as an email invitation with a weblink and was distributed to the Australian members of Endocrine Society of Australia (ESA), Australian and New Zealand Endocrine Surgeons (ANZES) and Australian Society of Otolaryngology, Head and Neck Surgery (ASOHNS). These organisations are the professional bodies of Australian endocrinologists, endocrine surgeons, and ear, nose and throat (ENT) surgeons, respectively. A reminder e‐mail was sent four weeks later to non‐responders.

### 2.3 Statistical analysis

Descriptive statistics were evaluated across clinical vignettes. Univariate and multivariable mixed effects regression models were used to examine the associations between management decisions (for each scenario, giving repeated measures at the clinician level), and clinician's demographic data and practice patterns. The model included fixed effects for the demographic and practice pattern variables, and a random intercept for the clinician to model the lack of independence induced by repeated measures of responses for each scenario. Variables were removed from the full model in a sequential manner, starting with the highest p‐value, provided their removal reduced the Akieke information criterion and did not alter the regression coefficients by more than 15%. Statistical analyses were programmed using SAS v9.4 (SAS institute, Cary, North Carolina, USA); alpha level of 0.05 was used for statistical significance.

## 3 RESULTS

### 3.1 Survey respondents

The survey was sent to 400 members of ESA, 123 members of ANZES and 488 members of ASOHNS. A total of 119 participants accessed the survey link, 109 answered at least 1 question and 87 completed at least 1 clinical scenario. It can be presumed that all 123 members of ANZES perform thyroid cancer surgery (response rate in this group of 24%) but it is unknown how many of the clinicians in other speciality groups are actively involved in thyroid cancer management. The data that support this article are available from the corresponding author upon request.

Demographic characteristics for the participants are included in Appendix 2. There was no statistically significant difference in gender, age or geographical location between respondents and non‐respondents (defined as those who opened the survey but did not complete any scenarios). However, there was a significant difference with responders reporting higher levels of MDT participation versus non‐responders (63% vs 38%, *p* = .04).

### 3.2 Initial management of WTDC

The summary of the participants' responses on the management of WDTC are presented in Table 2. Clinicians were asked to nominate their preferred operation for patients with low‐risk WDTC. The majority selected hemithyroidectomy for scenarios 2 and 3, 69% and 59%, respectively, while only 13% were comfortable with hemithyroidectomy with scenario 4. In scenario 2, 60% of surgeons would omit prophylactic central lymph node clearance, but 38% would perform a unilateral prophylactic central neck clearance; a higher proportion than the 26% who would offer total thyroidectomy. Despite there being no difference in the extent of surgery preferred by endocrine surgeons compared to ENT surgeons (76% vs 75% hemithyroidectomy, respectively) in this scenario; the approach of hemithyroidectomy with unilateral prophylactic central neck clearance was particularly prevalent amongst endocrine surgeons with 13 (50%) preferencing this approach compared with 2 (10%) of ENT surgeons.

**TABLE 2:** Characteristics of surgical decision‐making in the scenarios.

	Scenario 2 *n* = 87	Scenario 3 *n* = 75	Scenario 4 *n* = 71
Hemithyroidectomy	56 (69%)	N/A^a^	9 (13%)^b^
Total thyroidectomy	21 (26%)	N/A^a^	61 (87%)^b^
Active surveillance	2 (2.5%)	N/A^a^	N/A^a^
Clinician equipoise	2 (2.5%)	N/A^a^	N/A^a^
No further surgery	N/A^a^	44 (59%)	N/A^a^
Completion thyroidectomy	N/A^a^	16 (21%)	N/A^a^
Completion thyroidectomy and radioactive ablation	N/A^a^	15(20%)	N/A^a^
No prophylactic central neck dissection	48 (60%)	N/A^a^	16 (23%)
Yes (unilateral prophylactic central neck dissection)	30 (38%)	N/A^a^	39 (55%)
Yes (bilateral prophylactic central neck dissection)	2 (2.5%)	N/A^a^	16 (23%)
No radioiodine ablation	74(91%)	60 (80%)	27 (38%)
Yes, low dose (1 GBq, 25 mCI)	7 (8.6%)	15 (20%)	34 (48%)
Yes, high dose (4 GBq, 100 mCi)	N/A^a^	0	10 (14%)

*Note*: The patient in scenario 1 had an incidental, post‐operative diagnosis of micropapillary thyroid cancer; thus, there was no surgical decision‐making in this vignette.

^a^ Factor was not listed as an option in this scenario.

^b^ One missing.

For the higher risk scenario 4, the chosen management was more aggressive with only 23% of clinicians being comfortable to omit prophylactic central neck clearance, 55% would offer unilateral and 23% bilateral neck clearance. Endocrine surgeons were again more aggressive in their surgical approach to the central neck with only 4 (8%) electing to omit central neck clearance compared to 5 (42%) of ENT surgeons. When respondents were asked if they would offer radioactive iodine with the assumption that there is no biochemical or structurally persistent disease following surgery, 9% of clinicians in scenario 2, 20% in scenario 3% and 62% in scenario 4 would recommend radioactive iodine. In scenario 4, 48% would advise low dose (approx. 1 GBq, 25 mCi) and 14% would advise high dose (3–4 GBq, 100 mCi).

### 3.3 Follow‐up of WDTC

Clinicians were asked to indicate their preferred specialist care, follow‐up protocol for each scenario, assuming that all patients remained free of disease and that patients in scenarios 1, 2 and 4 had undergone a total thyroidectomy, while the patient in scenario 3 only underwent hemithyroidectomy. Of the 52 surgeons surveyed, 56% indicated that they would follow‐up the patients themselves, while the remainder would follow‐up in conjunction with or to refer back to an endocrinologist for follow‐up (these were included as specialist follow‐up). While 97% of respondents would offer long‐term follow‐up to the patient in scenario 4, 88%, 75% and 24%, respectively, would offer long‐term follow‐up to patients in scenario 3, 2 and 1. The five‐year mark was the most common time point for discharge to non‐specialist care; however, there was some preference towards 10‐year follow‐up in scenario 4 (Table 3). Univariate analysis showed that surgeons were significantly less likely to suggest specialist follow‐up for their patients for more than 5 years post‐operatively, *p* < .05.

**TABLE 3:** Intention, frequency and modality of follow‐up.

	Scenario 1 *n* = 87^b^	Scenario 2 *n* = 61	Scenario 3 *n* = 66	Scenario 4 *n* = 71^c^
*‘When would you discharge this patient back to their GP?’*				
Post‐operatively	*29 (33%)*	NA^a^	NA^a^	NA^a^
1 year after surgery	25 (29%)	*1 (1.6%)*	*1 (1.5%)*	*2 (2.8%)*
1–5 years after surgery	*32 (19%)*	*38 (63%)*	*41 (62%)*	*35 (49%)*
>5 years after surgery	*8 (9%)*	*22 (36%)*	*24 (36%)*	*34 (48%)*
*Modality of follow‐up*				
Ultrasound				
Not at all	3 (14%)	9 (15%)	3 (4.6%)	2 (2.9%)
6 monthly	1 (4.8%)	4 (6.6%)	10 (15%)	8 (12%)
12 monthly	13 (62%)	36 (59%)	43 (66%)	48 (70%)
24 monthly	2 (9.5%)	6 (9.8%)	7 (11%)	4 (5.8%)
Other	2 (9.5%	6 (9.8%)	2 (3.1%)	7(10%)
Thyroglobulin				
Not at all	1 (4.8%)	0	9 (14%)	0
6 monthly	4(19%)	15 (25%)	13 (20%)	22 (32%)
12 monthly	15 (71%)	45 (74%)	44 (67%)	46 (67%)
Other	1 (4.8%)	1 (1.6%)	0	1 (1.4%)

*Note:* Clinicians were asked to nominate follow‐up on the assumption that patients in Scenario and 1, 2, and 4 had undergone total thyroidectomy, while patient in Scenario 3 had only undergone hemithyroidectomy.

^a^ Factor was not listed as an option in this scenario.

^b^ Modality of follow‐up was only asked to participants who elected to follow‐up patients beyond 1 year in Scenario 1, there were 21 respondents.

^c^ 2 missing responses to modality of follow‐up.

The majority of the participants agreed that specialist follow‐up should be performed at 12 monthly intervals. Across all scenarios (Table 3), the majority of clinicians recommended 12 monthly neck ultrasound and serum thyroglobulin. A summary of factors which would prompt more intensive follow‐up are presented in Table 4. The common view was that larger tumour size, the presence of vascular invasion, an abnormal contralateral thyroid lobe after hemithyroidectomy, aggressive histopathology and positive BRAF V600E were factors that would increase the intensity of follow‐up.

**TABLE 4:** Factors leading to more intensive follow‐up.

Factors	Scenario 1 *n* = 86	Scenario 2 *n* = 80	Scenario 3 *n* = 73	Scenario 4 *n* = 68
Abnormal contralateral lobe (if patient chose hemithyroidectomy)	70 (81%)	57 (71%)	NA^a^	53 (78%)
Multi‐focal tumour	55 (64%)	47 (59%)	NA^a^	30 (44%)
BRAF 600E IHC positive	45 (52%)	44 (55%)	NA^a^	44 (65%)
Microscopic extra‐thyroidal extension	65 (76%)	59 (74%)	70 (96%)	39 (57%)
Vascular invasion^c^	80 (93%)	73 (91%)	71 (97%)	61 (90%)
Aggressive histological variant^c^	77 (90%)	71 (89%)	NA^a^	63 (93%)
Family History of thyroid cancer	47 (55%)	44 (55%)	31 (42%)	26 (38%)
Male gender	18 (22%)	17 (21%)	14 (19%)	NA^a^
Age >65 years	19 (17%)	26 (33%)	NA^a^	23 (34%)
Central neck nodal metastasis	NA^a^	NA^a^	NA^a^	62 (91%)
Larger tumour size^b^	8 vs 4 mm 6 (7%) 10–20 vs 4 mm 72 (84%)	30 vs 15 mm 59 (74%)	32 vs 18 mm 56 (77%)	>40 mm 55 (81%)

^a^ Factor was not listed as an option in this scenario.

^b^ Size was defined differently for each scenario.

^c^ These factors would increase papillary thyroid cancer to ATA intermediate risk.

### 3.4 Participant self‐evaluation of WDTC treatment

Clinicians were asked ‘do you feel that your patients' perception of their fear of recurrence equates to their actual risk profile of recurrence?’ . The majority of clinicians felt that 77% of patients had disproportionately high FCR, 19% were realistic and 4% feared recurrence less than the actual risk. Most clinicians (76%) felt that patients were reassured by their follow‐up. The intensity of follow‐up was deemed appropriate by 57% but the remaining 43% felt it was excessive. There was no significant difference in perceptions around FCR, the reassurance value of follow‐up or the appropriateness of follow‐up based on gender, speciality or MDT attendance. Review of free‐text comments suggested that some clinicians continue to follow‐up patients to manage their anxiety and fear of cancer recurrence. Others commented that their ability to discharge patients related to a lack of confidence that these patients would be well managed in a primary care setting.

### 3.5 WDTC treatment compared to clinician characteristics

Univariate and multivariable analysis was undertaken for treatment (total thyroidectomy as reference) and follow‐up (<5 years as reference). Parameters with *p* < .1 are displayed in Table 5; full data are provided in Appendix 3. There was no statistically significant difference in responses based on gender; geography (capital vs non‐capital city, state vs state); years in practice or volume of practice; clinician opinions of the appropriateness of follow‐up; or their perception of follow‐up as reassuring for patients. Both univariate and multivariable analysis showed that participants who attended a MDT were less likely to suggest total thyroidectomy for WDTC (*p* < .001 and *p* = .005, respectively). Univariate analysis suggested that surgeons were significantly less likely to suggest total thyroidectomy when compared to endocrinologists, OR 0.39, *p* < .05; however, these differences did not persist once multivariable analysis was undertaken.

**TABLE 5:** Univariable and multivariable analysis of treatment decision (total thyroidectomy) and follow‐up decision (>5 years follow‐up).

Variable	Univariate OR (95% CI)	Univariate *p* value	Multivariate OR (95% CI)	Multivariate *p* value
*Total thyroidectomy*				
MDT attendance	0.29 (0.15–0.54)	<.001	0.29 (0.12–0.68)	.005
Surgeon (endocrine or ENT)	0.39 (0.21–0.71)	.002	0.65 (0.33–1.25)	.19
*Follow‐up >5 years*				
MDT attendance	0.34 (0.10–1.11)	.072	0.23 (0.05–0.99)	.049
Surgeon (endocrine or ENT)	0.28 (0.09–0.93)	.037	0.35 (0.10–1.2)	.094

## 4 DISCUSSION

Since the publication of the 2015 ATA guidelines, there has been a move towards de‐escalation of treatment for low‐risk thyroid cancer. This study aimed to evaluate preferences for uptake of de‐escalation of initial management and follow‐up care of low‐risk WDTC by specialist clinicians in Australia. Our results suggest that many specialist thyroid cancer clinicians are comfortable to offer hemithyroidectomy and omit radioactive iodine for patients with low‐risk thyroid cancer, particularly where the primary tumour measures <2 cm and there are no higher risk features. This practice is consistent with international data confirming that in appropriately selected patients with low‐risk WDTC, oncological outcomes are not compromised with hemithyroidectomy.^14^

The philosophy behind de‐escalation is to minimise harm from overdiagnosis and the complications of treatment in those with low‐risk disease, while still maintaining excellent disease outcomes with appropriate treatment and surveillance for higher risk patients.^6^, ^15^ Overdiagnosis or overtreatment can result in costly use of unnecessary healthcare services and distress to patients and their support networks. There is evidence that complications such as vocal cord paralysis and hypoparathyroidism may be increased in lower‐volume settings; yet in this study, the uptake of de‐escalation was associated with MDT attendance, a likely surrogate marker of higher‐volume practice.^16^ Within the broader cancer field, MDTs have been associated with evidence‐based guideline awareness and application.^17^ It is probable that MDT attendance may also indicate exposure to discussion about de‐escalation and increased confidence with de‐escalation due to alignment with colleagues. The association between de‐escalation amongst surgeons in univariate analysis is interesting. Although it did not reach statistical significance in multivariable analysis, it is worthy of further investigation.

The high prevalence of prophylactic unilateral central neck clearance in association with hemithyroidectomy amongst endocrine surgeons in Scenario 2 is interesting. Neither the British Thyroid Association nor ATA Thyroid Cancer Guidelines recommend prophylactic unilateral central neck dissection in patients under the age of 45, with papillary thyroid cancer under 4 cm, with no other adverse features.^6^, ^18^ These guidelines, however, suggest that a personalised decision approach might be appropriate in patients who do not meet all these low‐risk criteria. It is hypothesised that unilateral central neck clearance might be preferred at the time of hemithyroidectomy to decrease rates of recurrence in the central neck and to obtain lymph node staging which might lead to increased rates of completion thyroidectomy and radioactive iodine treatment in this group of patients.^19^, ^20^ The hypothetical nature of these scenarios limits further investigation of prophylactic central lymph node clearance in the context of de‐escalated care; further research in this area is required.

Follow‐up for thyroid cancer aims to manage treatment side effects, detect recurrence early and provide supportive care to patients and their families. There is general consensus from this survey that the majority of the clinicians provide up to five years follow‐up post‐operatively for low‐risk WDTC. Long‐term follow‐up is associated with substantial cost to the healthcare with only a 2% risk of recurrence.^8^, ^9^ A recent Cochrane review suggested that there was little or no difference to overall survival with less intensive versus more intensive follow‐up.^21^ Interestingly, 43% of clinicians in this study felt that follow‐up protocols in their institution were excessive, yet 76% felt that patients were reassured by their follow‐up. Further study, including understanding patient perceptions of follow‐up and patient coping styles, is required to evaluate the true value of follow‐up in terms of clinical, psychosocial benefit and cost‐effectiveness.

The effect of de‐escalated care on HROoL remains unanswered with some studies suggesting that HRQoL is improved in patients treated with hemithyroidectomy.^22^, ^23^ In contrast, FCR has been shown to be a prominent driver of HRQoL detriments in WDTC,^24^, ^25^ and some studies have suggested that HRQoL is improved with total thyroidectomy due to a decrease in FCR with a more aggressive surgical approach.^22^, ^23^, ^26^ In this Australian study, 75% of clinicians felt that thyroid cancer survivors experienced FCR in excess of the true clinical risk of recurrence. This is in line with data from international patient studies, where FCR was found to be the most prominent concern amongst thyroid cancer survivors.^26^, ^27^ There are emerging data to suggest that clinician follow‐up visits provide substantial psychosocial support to patients and may decrease FCR in this group.^28^ There is also need for further research to investigate the effect of follow‐up on FCR and whether integrating interventions aimed at ameliorating FCR into clinician follow‐up may benefit patients.

The main weakness of this survey is the low response rate, with an overall response rate of 11.8% and only 87 of the 119 respondents completing at least one clinical scenario. Furthermore, the response rate for endocrine surgeons was 24%, for other specialists a clear response rate cannot be calculated; this is a potential source of selection bias in our analysis and limited our ability to analyse factors such as central neck dissection by speciality. Despite this low response rate, there were a large number of high‐volume clinicians in this survey, suggesting that the results may be broadly indicative of current practice in Australia. The definition and structure of MDT were not clearly defined in the questionnaire but left open to individual clinician interpretation; this is a possible source of heterogeneity in this study. Within Australia, there is no formal guidance regarding thyroid cancer MDT composition. However, the majority of MDT committees adhere to the standardised membership and governance as outlined in international guidelines.^29^ MDT in Australia is generally defined as a meeting with at least four medical practitioners from different medical specialities and includes allied health professionals. This broad definition reflects the flexibility required in cancer care due to the vast geography of Australia where MDTs must meet the needs of both metropolitan and regional cancer care centres.

The clinical vignettes were carefully designed to capture a variety of low‐risk thyroid cancer scenarios and were piloted by a group of surgeons and endocrinologists prior to wider dissemination. It is acknowledged that any group of scenarios cannot fully represent all the nuances of clinical care; particularly in the era of individualised patient care and shared decision‐making. Active surveillance was only listed as an option in Scenario 2 and was preferred by a minority of clinicians; we do not consider that this study has adequately assessed perceptions of active surveillance in the Australian setting. Finally, although clinical perceptions of FCR were surveyed, it is obviously critical that the patient perspective is fully understood. This is beyond the scope of this research but is the subject of other research by our group.

## 5 CONCLUSION

The uptake of de‐escalation (hemithyroidectomy and omission of radioactive iodine) in low‐risk thyroid cancer is likely to be commonplace in Australia, particularly by clinicians involved in thyroid cancer MDTs. Most patients are offered at least 5 years of follow‐up with annual review being most common. The majority of clinicians felt that patients' FCR was excessive, but that clinician follow‐up provided reassurance. These findings are critical to understanding patterns of thyroid cancer management within Australia and will be useful to any researcher wishing to study thyroid cancer survivorship.

## 6 AUTHOR CONTRIBUTIONS

**Winy Widjaja:** Formal analysis (equal); validation (supporting); writing – original draft (lead); writing – review and editing (equal). **Christopher Rowe:** Conceptualization (equal); data curation (equal); formal analysis (equal); investigation (equal); methodology (equal); project administration (equal); supervision (equal); validation (equal); writing – review and editing (equal). **christopher oldmeadow:** Formal analysis (lead); resources (equal); supervision (equal); writing – review and editing (equal). **Daron Cope:** Data curation (equal); investigation (equal); methodology (equal); project administration (equal); resources (equal); supervision (equal); writing – review and editing (equal). **Elizabeth fradgley:** Conceptualization (equal); data curation (equal); investigation (equal); resources (equal); supervision (equal); writing – review and editing (equal). **Christine Paul:** Conceptualization (equal); formal analysis (equal); investigation (equal); methodology (equal); supervision (equal); writing – review and editing (equal). **Christine O'Neill:** Conceptualization (equal); data curation (equal); formal analysis (equal); investigation (equal); methodology (equal); project administration (equal); resources (equal); supervision (lead); writing – original draft (supporting); writing – review and editing (lead).

## 8 CONFLICT OF INTEREST STATEMENT

None declared.

9

## Data Availability

The data that supports this article are available from the corresponding author upon request.

## APPENDIX 1 Confidential

### 1.1

Follow‐up of Patients with Well‐Differentiated Thyroid Cancer: a Survey of Clinician Preferences

25th August 2020

Dear Colleague,

It has recently been identified that patients with thyroid cancer may have significant quality of life detriments. We are exploring these issues in Australian patients and as part of this research we seek to understand the current treatment and follow‐up preferences of specialist clinicians in Australia.

This survey will explore current practice in the follow‐up of patients with well differentiated thyroid cancer through four case vignettes. It is not a test of your knowledge, but we do aim to explore your perception of risk of recurrence with each case and how this might influence your decisions in the follow‐up of individual patients. We hope to record a wide variety of responses. On average this survey will take 10 min to complete.

This survey is being sent to all Australian Members of the Endocrine Society of Australia, ANZ Endocrine Surgeons and the Australian Society of Otolaryngology, Head and Neck Surgery. If you treat any patients with thyroid cancer at any stage of their treatment, we would ask you to complete this study. It is important for our future research that we have has a high a participation rate as feasible. The results of this study will be used to inform future research in designing interventions to ameliorate quality of life detriments in patients with thyroid cancer. Research data from this study forms part of the PhD of Dr Christine O'Neill and will also be prepared for publication.

By completing this survey you are indicating your consent to participate in this project.

Participation is voluntary, responses will be anonymous. If you do not want to participate the close this window.

Survey responses once submitted cannot be reviewed, edited, or withdrawn.

Thank you and kind regards,

Dr Christine O'Neill Endocrine Surgeon

John Hunter Hospital and University of Newcastle, Newcastle NSW

Dr Christopher Rowe Endocrinologist

John Hunter Hospital and University of Newcastle, Newcastle NSW

Dr Daron Cope 23/02/2021 8:59

ENT Surgeon

John Hunter Hospital and University of Newcastle, Newcastle NSW

This research has been approved by the Hunter New England Human Research Ethics Committee of Hunter New England Local Health District, reference 2020/ETH01892.

Should you have concerns about your rights as a participant in this research, or you have a complaint about the manner in which the research is conducted, it may be given to the researcher, or, if an independent person is preferred, to Dr Nicole Gerrand, Manager Research Ethics and Governance, HNE Research Office Level 3, POD, Hunter Medical Research Institute, Lot 1 Kookaburra Circuit, New Lambton Heights NSW 2305, telephone (02) 49214950, email HNELHD-HREC@health.nsw.gov.au

## APPENDIX 2 Clinicians demographic and practice characteristics

### 2.1

**Table:**

Demographic	Category	Participants who completed at least one scenario (%) (*N* = 87)	Participants who did not complete any scenarios (%) (*N* = 22)	*p*‐Value
Age (years) *n* = 107	<45	37 (43%)	8 (38%)	.82
	46‐55	31 (36%)	8 (38%)	
	55‐65	13 (15%)	3 (14%)	
	>65	5 (5.8%)	2 (9.5%)	
Gender *n* = 107	Male	48 (56%)	11 (52%)	.835
Medical Speciality	Endocrinologist	40 (46%)	12 (55%)	.002
	Endocrine (General) surgeon	28 (32%)	2 (9.1%)	
	ENT, head and neck surgeon	18 (21%)	4 (18%)	
	Other	1 (1.1%)	4 (18%)	
Practice experience (years) *n* = 108	1–5	16 (18%)	6 (29%)	.60
	6–10	17 (20%)	2 (9.5%)	
	11–15	18 (21%)	4 (19%)	
	>15	36 (41%)	9 (43%)	
Volume of WDTC cases (per year) *n* = 108	<10	26 (30%)	11 (52%)	.23
	10–20	30 (34%)	6 (29%)	
	21–50	19 (22%)	3 (14%)	
	>50	12 (14%)	1 (4.8%)	
MDT attendance *n* = 107	Yes	45 (63%)	8 (38%)	.04
Geography *n* = 102	Capital city	56 (68%)	16 (80%)	.30

## APPENDIX 3 Univariate and multivariable analysis of treatment decision (total thyroidectomy) and follow‐up decision (>5 years follow‐up)

### 3.1

**Table:**

Variable	Univariate OR (95% CI)	Univariate *p* value	Multivariate OR (95% CI)	Multivariate *p* value
*Total thyroidectomy*				
MDT attendance	0.29 (0.15–0.54)	<.001	0.29 (0.12‐0.68)	.005
Surgeon (endocrine or ENT)	0.39 (0.21–0.71)	.002	0.65 (0.33–1.25)	.19
Female	1.80 (0.95–3.43)	.073	1.21 (0.62–2.34)	.58
Case volume per year >10 patients	0.57	.108	1.34	.484
Practicing Experience of >10 years	0.76	.418	1.51	.251
*Follow‐up >5 years*				
MDT Attendance	0.34 (0.10–1.11)	.072	0.23 (0.05–0.99)	.049
Surgeon (endocrine or ENT)	0.28 (0.09–0.93)	.037	0.35 (0.10–1.2)	.094
Female	3.11 (0.92–10.54)	.069	2.16 (0.64–7.23)	.21
Case volume per year >10 patients	0.98	.975	2.49	.251
Practicing Experience of >10 years	1.64	.433	2.37	.239
